# Hemolytic Uremic Syndrome Presenting With Acute Renal Failure in an Adult: A Case Report

**DOI:** 10.7759/cureus.90954

**Published:** 2025-08-25

**Authors:** Michal Gajewski, Maksym Gmur, Weronika Goliat, Konrad Haraziński, Izabela Jastrzebska, Barbara Sławińska, Oliwia Sysło, Nikola Rubik, Zuzanna Błecha, Nicole Maryniak

**Affiliations:** 1 Medicine, Provincial Hospital in Poznań, Poznań, POL; 2 Internal Medicine, Międzyleski Specialist Hospital in Warsaw, Warsaw, POL; 3 Medicine, Medical University of Silesia in Katowice, Katowice, POL; 4 Faculty of Medicine, Academy of Silesia, Katowice, POL; 5 Medicine, Academy of Silesia, Katowice, POL; 6 Faculty of Medicine, Medical University of Silesia in Katowice, Katowice, POL; 7 Medicine, Zagłębiowski Oncology Center in Dąbrowa Górnicza, Dąbrowa Górnicza, POL

**Keywords:** acute renal failure, adult, hemolytic uremic syndrome, plasma exchange, thrombotic microangiopathy

## Abstract

Hemolytic uremic syndrome (HUS) is a rare but life-threatening thrombotic microangiopathy in adults, classically characterized by the triad of microangiopathic hemolytic anemia, thrombocytopenia, and acute kidney injury. While pediatric HUS is commonly associated with Shiga toxin-producing *Escherichia coli*, adult presentations are often atypical and carry a higher risk of progression to chronic kidney disease or death. We present the case of a 49-year-old woman with acute renal failure and features of HUS, managed with hemodialysis and plasma exchange, emphasizing the diagnostic and therapeutic challenges in adult HUS.

## Introduction

Hemolytic uremic syndrome (HUS) is a rare thrombotic microangiopathy (TMA) [1,2] defined by the triad of non-immune microangiopathic hemolytic anemia, thrombocytopenia, and acute kidney injury (AKI). HUS can be broadly classified into typical (Shiga toxin-associated) and atypical (complement-mediated) forms. Typical HUS, commonly due to Shiga toxin-producing *Escherichia coli* (STEC), predominantly affects children and follows a prodrome of diarrhea, often bloody. In contrast, atypical HUS (aHUS) [3,4] accounts for approximately 5%-10% of cases and is associated with complement dysregulation, often triggered by infections, drugs, autoimmune disease, or pregnancy, and carries a poor renal prognosis.

The underlying pathogenesis involves toxin- or complement-mediated injury to vascular endothelial cells, particularly in the renal microvasculature. This results in platelet activation, microthrombi formation, and mechanical destruction of red blood cells, as they traverse narrowed vessels, ultimately producing the triad of hemolytic anemia, thrombocytopenia, and renal impairment [1,2].

Renal involvement is the hallmark of HUS and ranges from transient AKI to irreversible end-stage renal disease [5,6]. Up to 50% of adult patients may require dialysis during the acute phase, and mortality remains high despite supportive care. Prompt recognition and differentiation [4,6] from other TMAs, such as thrombotic thrombocytopenic purpura (TTP), are critical to initiating appropriate therapy, including plasma exchange and, in the case of aHUS, complement inhibition with eculizumab [3,4,7].

While pediatric HUS has been extensively described, reports of adult-onset disease remain relatively scarce, and its clinical course is often more severe and less predictable. By presenting this case, we aim to highlight its diagnostic and therapeutic challenges, contributing to the growing literature on adult HUS and supporting earlier clinical recognition and timely management in similar patients.

## Case presentation

A 49-year-old previously healthy woman developed abdominal pain for five days, followed by diarrhea for two days. She initially received outpatient treatment with trimethoprim-sulfamethoxazole, nifuroxazide, and loperamide. Her symptoms persisted, and she presented to a hospital, where laboratory evaluation revealed AKI (creatinine: 5.5 mg/dL), severe anemia with thrombocytopenia, and elevated bilirubin and lactate dehydrogenase (LDH). She received two units of packed red blood cells (PRBCs) and was subsequently transferred to the nephrology department for further management. On admission, the patient appeared moderately ill, complaining of profound weakness, with anuria but no ongoing diarrhea. Laboratory tests showed microangiopathic hemolytic anemia with schistocytes and elevated LDH, thrombocytopenia, and acute renal failure. A diagnosis of HUS with acute renal failure was established. Complement studies and genetic testing for aHUS were not performed during hospitalization due to limited availability and the acute need for supportive therapy. She underwent urgent hemodialysis via a temporary central venous catheter and subsequently received eight sessions of plasma exchange with 3 L of plasma replacement per session. Due to persistent anemia, she required 10 units of PRBCs during hospitalization. In the following days, the temporary catheter was replaced with a permanent right internal jugular catheter for ongoing dialysis therapy. Following clinical improvement, she was discharged home for outpatient dialysis and nephrology follow-up. Compared with other reported adult cases, our patient’s presentation with rapid progression to dialysis dependence is consistent with the high morbidity described in the literature, where up to 30% of adults progress to mortality and more than one-quarter require long-term dialysis [6]. Similar cases of diarrhea-associated HUS in adults have also demonstrated a more severe course than in pediatric patients, with higher rates of transfusion and renal replacement therapy [1,2]. The chronological progression of the patient’s symptoms, interventions, and clinical course is summarized in Table 1. Table 2 summarizes the dynamic changes in key laboratory parameters at admission, during hospitalization, and at discharge.

**Table 1:** Clinical timeline of the patient This table summarizes the chronological sequence of the patient’s symptoms, interventions, and clinical outcomes during hospitalization. Cr: Creatinine; Hb: Hemoglobin; IJ: Internal jugular vein; Plt: Platelet count; PRBC: Packed red blood cell; TPE: Therapeutic plasma exchange

Hospital day	Clinical events/interventions	Key findings/outcomes
Day 5	Onset of abdominal pain	No prior medical history
Day 3	Onset of watery diarrhea	Self-treated with biseptol, nifuroxazide, and loperamide
Day 1	Admitted to the hospital	Cr: 5.5 mg/dL, Hb: ~7 g/dL, Plt: 40k; 2U PRBCs transfused
Day 0	Transferred to the nephrology department	Anuric; peripheral smear with schistocytes and acanthocytes
Day 1	Temporary dialysis catheter placed; first hemodialysis	Initiation of renal replacement therapy
Day 2	Therapeutic plasma exchange initiated	3 L plasma/session; daily TPE
Day 2-10	Eight sessions of plasma exchange completed	Progressive hematologic improvement
Day 5-15	Multiple PRBC transfusions (10U total)	Stabilization of Hb: ~9-10 g/dL
Day 16	Permanent right IJ dialysis catheter placed	Patient remains dialysis dependent
Discharge	Transferred for outpatient hemodialysis	Clinically stable, follow-up arranged

**Table 2:** Laboratory findings of the patient at admission and during hospitalization This table presents the patient’s key laboratory values at admission, at their peak or lowest levels during hospitalization, and at discharge, compared with reference ranges. LDH: Lactate dehydrogenase

Parameter	Admission	Peak/lowest	At discharge	Reference range
Hemoglobin (g/dL)	7.2	6.8	9.5	12–16
Platelets (×10³/µL)	42	35	120	150–450
LDH (U/L)	1800	2200	600	135–225
Creatinine (mg/dL)	5.5	7.8	6.2	0.6–1.1
Bilirubin (mg/dL)	2.1	3.0	1.2	0.2–1.0

## Discussion

HUS in adults is rare and associated with severe morbidity [1,2,6]. Its differential diagnosis includes typical STEC-HUS, aHUS [3,4,7], TTP, and secondary TMAs. Typical HUS follows Shiga toxin-induced endothelial injury [1,2], while aHUS involves complement dysregulation. TTP is distinguished by severe ADAMTS13 deficiency [3,7] and more prominent neurologic features. Secondary TMAs may result from autoimmune disease [3,6], pregnancy, infections, or medications. Unlike pediatric STEC-HUS, where complete renal recovery is more frequent, adult-onset cases such as ours are more likely to progress to chronic kidney disease, underscoring the prognostic gap between age groups [5,6]. Pathophysiology involves endothelial damage, platelet aggregation, and microthrombi formation in renal arterioles and capillaries, causing microangiopathic hemolytic anemia, thrombocytopenia, and acute renal injury [1,2]. Adult HUS carries a worse prognosis [5,6] than pediatric cases, with 20%-40% developing chronic kidney disease or requiring prolonged dialysis. In our patient, early initiation of dialysis and plasma exchange led to clinical improvement, consistent with supportive management strategies. Long-term follow-up is essential due to the risk of chronic kidney disease and recurrence in some adult HUS cases. In contrast to the catastrophic progression described by other authors, where aHUS rapidly led to multiorgan involvement and poor outcomes, our patient demonstrated hematologic stabilization after plasma exchange, highlighting the variability of adult HUS trajectories [4].

As outlined in Table 3, the prognosis of adult HUS remains guarded, with mortality reported in approximately 30% of cases. The high frequency of dialysis dependence (27%) reflects the severity of renal injury observed in this population and is mirrored in our patient, who required ongoing hemodialysis at discharge. Although progression to chronic renal failure occurs in a smaller proportion of patients (14%), this finding highlights the potential for long-term morbidity and the need for close nephrology follow-up. Furthermore, the recurrence rate of 24% underscores the unpredictable disease trajectory and reinforces the importance of long-term monitoring even after apparent clinical recovery. Table 4 provides an integrated overview of the patient’s laboratory parameters in relation to key clinical events, highlighting how interventions such as dialysis, plasma exchange, and transfusions corresponded with hematologic and renal recovery.

**Table 3:** Reported outcomes in adult HUS This table outlines the reported frequencies of major outcomes in adult patients with hemolytic uremic syndrome, including mortality, dialysis requirement, chronic renal failure, and recurrence [6].

Outcome	Frequency (%)
Mortality	30
Dialysis requirement	27
Chronic renal failure	14
Recurrence	24

**Table 4:** Consolidated clinical timeline and laboratory results This consolidated table integrates the patient’s clinical course with corresponding laboratory trends, allowing quick correlation between interventions (dialysis, plasma exchange, and transfusions) and hematologic/renal recovery. IJ: Internal jugular; LDH: Lactate dehydrogenase; PRBC: Packed red blood cell

Hospital day	Clinical events/interventions	Hemoglobin (g/dL)	Platelets (×10³/µL)	LDH (U/L)	Creatinine (mg/dL)	Bilirubin (mg/dL)
Day 5	Onset of abdominal pain	–	–	–	–	–
Day 3	Watery diarrhea; self-treated	–	–	–	–	–
Day 1	Hospital admission; 2U PRBC transfused	~7.0	40	↑	5.5	↑ (2.1)
Day 0	Transfer to nephrology; anuric; schistocytes on smear	7.2	42	1800	5.5	2.1
Day 1	Dialysis catheter placed; first hemodialysis	–	–	–	–	–
Day 2-10	Eight sessions of plasma exchange (3 L/session)	Progressive rise	Progressive rise	↓	↓	↓
Day 5-15	10U PRBC transfused in total	9-10	–	–	–	–
Day 16	Permanent right IJ catheter placed	–	–	–	–	–
Discharge	Outpatient dialysis; clinically stable	9.5	120	600	6.2	–

## Conclusions

This case highlights the importance of prompt recognition and syndromic treatment of HUS in adults presenting with TMA features and renal failure. Early plasma exchange and dialysis support remain the mainstay of initial therapy. Distinguishing typical from aHUS is critical for long-term management, including consideration of complement inhibition. Although complement blockade with agents such as eculizumab has emerged as a cornerstone of therapy for aHUS, this option was not available in our center during the patient’s treatment, which limited therapeutic possibilities. This case, therefore, underscores both the need for timely diagnosis and the challenges posed by restricted access to targeted therapies.
